# Supplementary material for: The interactome of intact mitochondria by cross-linking mass spectrometry provides evidence for coexisting respiratory supercomplexes
Source: Mol Cell Proteomics. 2017 Dec 8;17(2):216–32. doi: 10.1074/mcp.RA117.000470 (PMC5795388; doi:10.1074/mcp.RA117.000470)
Supplement: Supplemental Data [file supp_RA117.000470_133922_0_supp_23978_fzffwf.zip › spectra_annotation/mito_spectra_annotation/422-1-18-1-11-1.pdf]

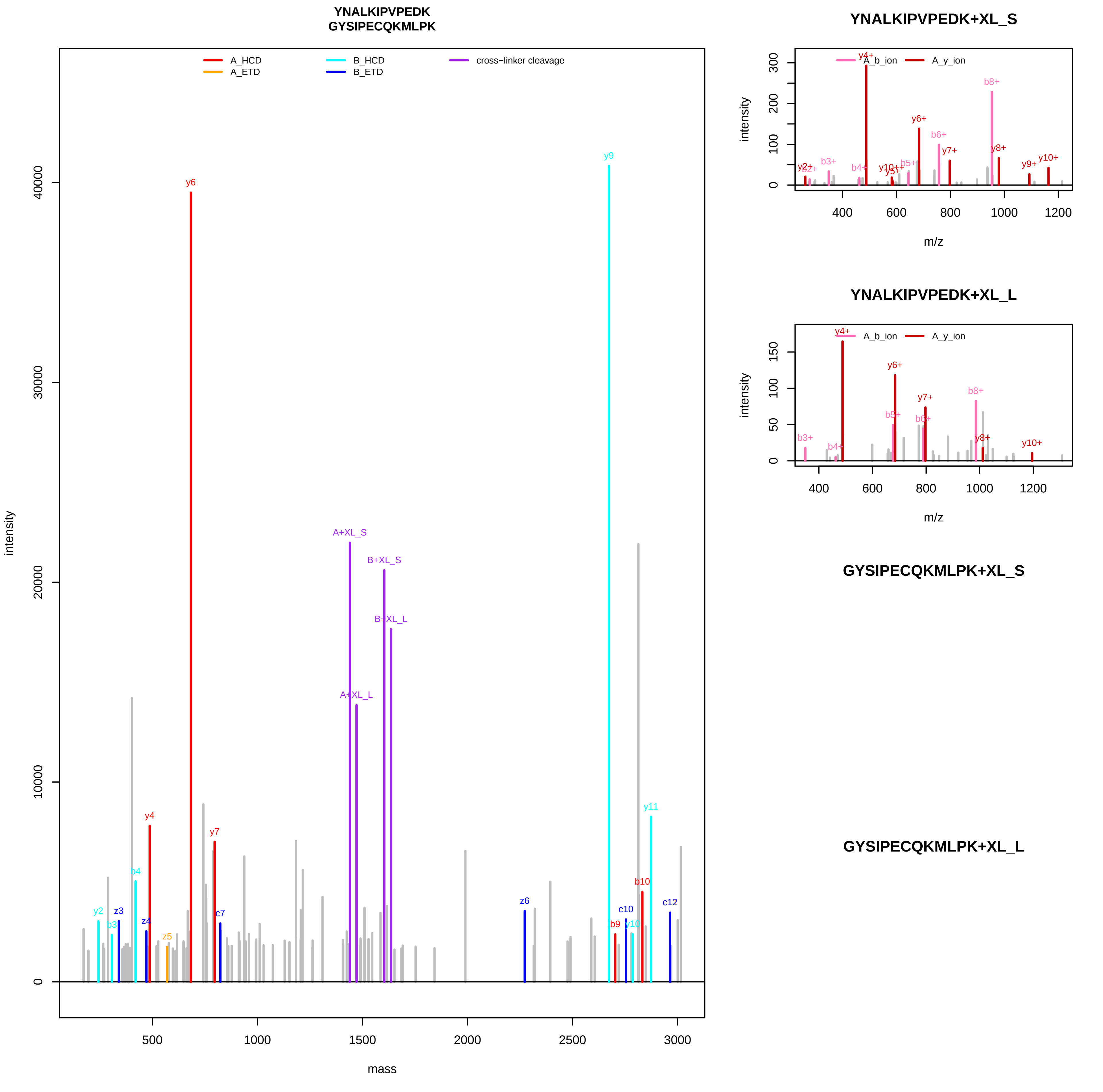

YNALKIPVPEDK
GYSIPECQKMLPK
A_HCD
A_ETD
B_HCD
B_ETD
cross−linker cleavage
intensity
mass
0
10000
20000
30000
40000
500
1000
1500
2000
2500
3000
y6
y9
A+XL_S
B+XL_S
B+XL_L
A+XL_L
y4
y7
y11
b4
b10
z6
c12
c10
y2
z3
b3
z4
z5
c7
b9
y10
YNALKIPVPEDK+XL_S
A_b_ion
A_y_ion
m/z
y4+
b8+
y6+
b6+
y7+
y8+
y10+
y9+
b3+
b4+
b5+
y5+
y10++
y2+
b2+
YNALKIPVPEDK+XL_L
GYSIPECQKMLPK+XL_S
GYSIPECQKMLPK+XL_L
